# Risk Factors Leading to Anti-TNF Alpha Therapies in Pediatric Severe Uveitis

**DOI:** 10.3389/fped.2022.802977

**Published:** 2022-03-04

**Authors:** Delphine Osswald, Anne-Cécile Rameau, Joëlle Terzic, Christelle Sordet, Tristan Bourcier, Arnaud Sauer

**Affiliations:** ^1^Service d'Ophtalmologie, Hôpitaux Universitaires de Strasbourg, Strasbourg, France; ^2^Service de Pédiatrie, Hôpitaux Universitaires de Strasbourg, Strasbourg, France; ^3^Service de Rhumatologie, Hôpitaux Universitaires de Strasbourg, Strasbourg, France

**Keywords:** uveitis (MeSH), anti-TNF agent, children, risk factors, juvenile idiopathic arthritis associated uveitis, idiopathic uveitis in children

## Abstract

**Purpose:**

Pediatric uveitis is the leading cause of acquired child blindness, due to unremitting inflammation and long-term steroid exposition. Biotherapies with anti-tumor necrosis factor alpha (anti-TNFα) are effective in controlling inflammation for severe pediatric uveitis in recent studies. Major concern of anti-TNFα prescription is the balance between the severity of the disease and side effects of the drug. The aim of the present study is to describe a cohort of children with severe uveitis and to highlight the risk factors for a pejorative development that led to the prescription of anti-TNFα drugs.

**Method:**

A retrospective case-control study was carried out on children with uveitis associated with systemic inflammatory disease or idiopathic uveitis, with a minimum follow-up of 5 years. Anti-TNFα-treated patients (case) were studied and compared with patients who were not requiring anti-TNFα (control). Univariate logistic regression analyses were performed to compare both groups and determine the risk factors for anti-TNFα therapy.

**Results:**

Seventy-three cases of pediatric uveitis were included, 13 cases and 60 controls. The risk factors associated with increased odds of anti-TNFα therapy were initial systemic disorder associated with uveitis [OR = 11.22 (1.37–91.85), *p* = 0.0241), family history of autoimmune diseases [OR = 9.43 (2.27–39.15), *p* = 0.0020], uveitis diagnosis before the age of 6 [OR = 4.05 (1.16–14.13), *p* = 0.0284], eye surgery [OR = 26.22 (2.63–261.77), *p* = 0.0054], ocular complications at the first slit lamp exam [OR = 67.11 (3.78–1191.69), *p* = 0.0042], low visual acuity at diagnosis (≥0.3 logMAR) [OR = 11.76 (2.91–47.62), *p* = 0.0005] and especially low binocular acuity at diagnosis (≥0.3 logMAR) [OR = 8.75 (1.93–39.57), *p* = 0.0048], panuveitis [OR = 9.17 (2.23–37.60), *p* = 0.0021], having positive ANA [OR = 3.89 (1.07–14.11), *p* = 0.0391], and positive HLA B27 [OR = 9.43 (2.27–39.16), *p* = 0.0020].

**Conclusion:**

Those risk factors could be used to establish a new follow-up and treatment schedule for severe uncontrolled uveitis. This could help to better predict the best time to start anti-TNF therapy.

## Introduction

Pediatric uveitis is the leading cause of acquired child blindness with a prevalence of 30 cases per 100,000 inhabitants. It is a rare disease, with about 500 new cases per year in France, but stakes are huge. The main causes of childhood uveitis are systemic inflammatory diseases (5–47%) followed by infectious etiologies (2–39%). In a relatively large part (25–60% of cases depending on series), clinical and biological work-up does not allow to find the causes of the ocular inflammation: It is then an idiopathic uveitis (1–4). Treatment differs depending on the etiologies. Inflammatory uveitis is treated in the acute phase with corticosteroids (topically or systemically, depending on the severity) (5, 6). For all chronic cases, damage is due to unremitting inflammation and long-term steroid exposition. That is why, controlling eye inflammation and sparing corticosteroids are major issues in pediatric uveitis management (7, 8).

Concerning corticosteroid sparing, there is no established guideline to an appropriate therapeutic behavior, and therapeutic decision is often guided by the personal experiences of the ophthalmologist and the pediatrician (9–11). Methotrexate and more recently anti-tumor necrosis factor alpha (TNFα) have allowed a marked improvement in the functional prognosis of severe inflammatory pediatric uveitis, as demonstrated in the *ADJUVITE* and *SYCAMORE* trials (12, 13). Prescribing anti-TNFα treatment raises two main questions. The first is the choice of the molecule, according to the expected efficacy on ocular and associated damage and acceptability for families (routes of administration and periodicity). The second is the optimal time to start treatment. Treatment should be started early enough to prevent complications, but in a reasonable way to limit side effects. The identification of initial factors of severity and/or of pejorative evolution would, thus, be very useful to guide the prescription of anti-TNFα drugs.

The aim of the present study is to describe a cohort of children with severe uveitis associated with inflammatory or idiopathic diseases and to highlight the risk factors for a pejorative development that led to the prescription of anti-TNFα drugs.

## Methods

### Design

A retrospective case-control study was carried out in Strasbourg University Hospital (France). From 2000 to 2018, the inclusion criteria were the presence of related inflammatory disease or idiopathic chronic symptomatic uveitis (persistent uveitis with relapse in <3 months after discontinuing treatment) in a child (age at onset >13 and age <18 years at the end of the follow-up) with a minimum follow-up of 5 years. For all the included patients (presenting with chronic uveitis resistant to first-line local treatment, the treatment consisted of systemic corticotherapy started at 1 mg/kg/day for 2 weeks. Methotrexate (dose appropriate for the weight of the child) was initiated in all patients in the study. Clinical data and uveitis classification were based on the SUN Working Group recommendations (14). Recruitment was carried out using internal hospital databases as well as health insurance data. Infectious uveitis, posttraumatic, demyelinating diseases, postsurgical inflammation, and masquerade syndrome were excluded.

According to the clinical course, patients included were then separated into two groups. The group of cases was defined by chronic uveitis requiring the prescription of anti-TNFα (etanercept, infliximab, or adalimumab) due to insufficient control of inflammation and corticosteroid dependence (need for corticosteroid therapy >5 mg per day after a 3-month period of methotrexate or greater than one drop per day for a period of more than 1 month). Controls were patients with an objective uveitis detected at the slit lamp examination and who did not required anti-TNFα therapy during the 5-year follow-up. The clinical and biological data of the cases and controls were then compared in order to define the elements favoring the prescription of an anti-TNFα treatment. The protocol was approved by the Local Ethics Committee (Strasbourg, France) in accordance to the tenets of the Declaration of Helsinki.

### Collected Data

Demographic data (gender, date of birth, age at diagnosis), patient history (personal and familial medical conditions), initial ophthalmological state (initial visual acuity, intraocular pressure, Tyndall effect, keratic precipitates, posterior segment abnormalities), ocular complications [posterior synechiae, cataract, band keratopathy, optic disc edema, cystoid macular edema (macular thickness >320 μm with inner retinal cysts), vasculitis, retinochoroiditis lesions], characteristics of uveitis (uni- or bilateral; acute, recurrent, or chronic evolution; anterior, posterior, intermediate, or panuveitis), etiologic assessment data [full blood count (FBC), erythrocyte sedimentation rate (ESR), C-reactive protein (CRP), kidney and liver function, serum angiotensin-converting enzyme (ACE), antinuclear antibodies (ANA), rheumatoid factor (RF), antineutrophil cytoplasmic antibodies (ANCA), anti-DNA, complement test, serum immunoglobulin count, HLA B27, herpes simplex virus (HSV)–Toxoplasma–Toxocara–varicella-zoster virus (VZV) serologies, chest x-ray, tuberculin skin test, pulmonary function testing depending on the clinic presentation] and treatments in place and their introduction sequences were screened. Patients had a clinical examination every 3 months with systematic collection of data from slit lamp examination, the fundus, and OCT. Biological data were collected at least during the initial assessment and then every 2 years (three collections per patient). Data were retrospectively collected in the digital files of patients (*Dx-Care*®) for the newest cases and in paper files for the oldest.

### Statistical Analysis

We examined distributions for all variables of interest by determining the frequencies, means, and measures of variance. To evaluate the statistical significance of the unadjusted associations between case (anti-TNFα treatment) and control (no anti-TNFα treatment) status and the characteristics of the participants, we used either Fisher's exact tests or Pearson's chi-square tests for categorical variables. We used unconditional logistic regression to compute adjusted odds ratios (ORs) and 95% confident interval (CI) of independent variables. A value of *p* < 0.05 was determined to be significant. The statistical software used was GraphPad Prism 5 (GraphPad Software Inc., San Diego, CA, USA).

## Results

Seventy-three patients with pediatric uveitis were included, and among them were 33 uveitis related to systemic inflammatory disease (45%) and 40 idiopathic uveitis (55%). Because of uncontrolled eye inflammation, treatment by anti-TNFα was decided for 13 (18%) patients (cases). Of the patients, 60 (82%) remained stable without anti-TNFα (controls). Demographic features were similar in both patient groups (Table 1). The repartition of the etiologies in each group is described in Table 2.

**Table 1:** Demographic and clinical data for the 13 anti-tumor necrosis factor alpha (TNFα)-treated patients (Case) and the 60 patients without anti-TNFα (control).

**Demographic and clinical data**	**Anti-TNFα-treated patients (Case, *N* = 13)**	**Patients without Anti-TNFα (Control, *N* = 60)**
Gender female *N* (%)	8 (61%)	31 (52%)
Mean age of diagnose (years)	7	8
Unilateral uveitis *N* (%)	5 (38%)	33 (55%)

**Table 2:** Etiologic data for the 13 anti-TNFα treated patients (cases) and the 60 no anti-TNFα patients (control).

**Etiologies**	**Anti-TNFα-treated patients (cases,** ***N*** **= 13)**	**Patients without anti-TNFα (controls,** ***N*** **= 60)**
**Inflammatory uveitis**	9	24
Juvenile Idiopathic Arthritis	5	9
Sarcoidosis	0	4
Ankylosing Spondylitis	3	6
Lupus	1	0
Behçet disease	0	1
Blau syndrome	0	1
TINU syndrome	0	2
Kawasaki	0	1
**Idiopathic uveitis**	4	36

Only 23 patients (31%) were already known for having a systemic disease that could lead to uveitis. The mean duration of uveitis evolution before the introduction of anti-TNFα was 16 months. The mean duration of oral steroid impregnation over the 5-year follow-up was 22.4 months.

Comparative data with univariate ORs for the risk factors relative to the 13 anti-TNFα-treated patients (case) and 60 patients without anti-TNFα (control) are provided in Table 3.

**Table 3:** Comparative data with univariate ORs for the risk factors relative to the 13 anti-TNFα-treated patients (case) and 60 patients without anti-TNFα (control).

**Treatment by anti TNFα- related risk factors**	**Patients in case group (%)**	**Patients in control group (%)**	**OR (95% confidence interval)**	***p***
**Risk factors related to the etiology**				
Juvenile idiopathic arthritis	38	15	3.54 (0.94–13.29)	0.0610
Idiopathic	31	51	0.42 (0.11–1.49)	0.1797
Ankylosing spondylitis	23	8	2.70 (0.69–11.11)	0.1529
Lupus	8	0	13.7 (0.53–354.1)	0.1117
**Risk factors relative to the patient**				
Initial systemic disorder associated with uveitis	92	51	11.22 (1.37–91.85)	0.0241
History of autoimmune diseases in relatives	46	8	9.43 (2.27–39.15)	0.0020
Male	38	47	0.71 (0.21–2.44)	0.5910
Female	62	52	1.02 (0.31–3.39)	0.9732
Diagnoses ≤ 6 years old	62	28	4.05 (1.16–14.13)	0.0284
Inaugural uveitis	69	75	0.75 (0.20–2.79)	0.6680
Eye surgery	31	2	26.22 (2.63–261.77)	0.0054
**Risk factors related to complications**				
Initial complications	100	61	67.11 (3.78–1191.69)	0.0042
Papillary oedema	31	33	0.89 (0.24–3.24)	0.8584
Macular oedema	69	17	11.25 (2.89–43.81)	0.0005
Cataract	38	5	11.87 (2.37–59.49)	0.0026
Band keratopathy	31	3	12.89 (2.05–80.90)	0.0064
Synechia	61	34	3.20 (0.92–11.05)	0.0659
Risk factors relative to uveitis	77	28	11.76 (2.91–47.62)	0.0005
Low visual acuity at diagnosis (≥0.3 logMAR or 20/40)				
Low binocular acuity at diagnosis (≥0.3 logMAR or 20/40)	38	7	8.75 (1.93–39.57)	0.0048
Bilaterality	69	46	1.83 (0.54–6.24	0.3350
Anterior uveitis	23	52	0.28 (0.07–1.12)	0,0724
Posterior uveitis	8	13	0.54 (0.06–4.75)	0.5800
Intermediate uveitis	0	5	0.61 (0.03–12.49)	0.7473
Panuveitis	77	27	9.17 (2.23–37.60)	0.0021
**Risk factors relative to etiological assessment**				
ANA positive	69	37	3.89 (1.07–14.11)	0.0391
HLA B27 positive	46	8	9.43 (2.27–39.16)	0.0020
FR positive	23	7	4.20 (0.81–21.68)	0.0866

The risk factors associated with the greatest increased odds of anti-TNFα therapy were as follows: initial systemic disorder associated with uveitis [OR = 11.22 (1.37–91.85), *p* = 0.0241], family history of autoimmune diseases [OR = 9.43 (2.27–39.15), *p* = 0.0020], uveitis diagnosis before the age of 6 [OR = 4.05 (1.16–14.13), *p* = 0.0284], eye surgery [OR = 26.22 (2.63–261.77), *p* = 0.0054], and ocular complications at the first slit lamp exam [OR = 67.11 (3.78–1191.69), *p* = 0.0042]. Among those complications, some were more likely to be associated with anti-TNFα treatment: macular edema [OR = 11.25 (2.85–43.81), *p* = 0.0005], cataract [OR = 11.87 (2.37–59.49), *p* = 0.0026], and band keratopathy [OR = 12.89 (2.05–80.90 *p* = 0.0064]. Low visual acuity at diagnosis (≥0.3 logMAR) [OR = 11.76 (2.91–47.62), *p* = 0.0005] and especially low binocular acuity at diagnosis (≥0.3 logMAR) [OR = 8.75 (1.93–39.57), *p* = 0.0048] were associated to a higher need of biotherapy. Panuveitis seemed to be more often associated with anti-TNFα treatment [OR = 9.17 (2.23–37.60), *p* = 0.0021]. Finally, having positive ANA [OR = 3.89 (1.07–14.11), *p* = 0.0391] and positive HLA B27 [OR = 9.43 (2.27–39.16), *p* = 0.0020] seemed to increase the risk of having an anti-TNFα therapy.

## Discussion

This study made it possible to collect the files of 73 children with chronic anterior uveitis with a minimum follow-up of 5 years. The prolonged follow-up of children with chronic uveitis is relatively rare in the literature. As uveitis is a rare disease, a retrospective recruitment was preferred, although bias are possible (notably missing data). The comparative analysis of the clinical and laboratory data of the cases and of the controls made it possible to highlight factors increasing the risk of resorting to anti-TNF immunotherapy. Moreover, the collection of data, having been carried out before the prescription of anti-TNFα, allows a quantification of the evolution risk of the various factors present from the inclusion consultation.

Results match with common knowledge in this field. For example, JIA is more associated with anti-TNFα therapy than other etiologies because diagnosis is often made very late due to the limited and discrete symptoms and clinical signs in children. Prescribing habits in JIA uveitis are quite easily oriented toward anti-TNFα studies having shown an important efficacy of this treatment (8, 15). Positive ANA is also associated with anti-TNFα therapy [OR = 3.89 (1.07–14.11), *p* = 0.0391], and it is established that they are a risk factor of developing uveitis when having JIA (16, 17). According to Zuber et al., antirheumatic drugs (DMARDs) and corticosteroids were more frequently clinically ineffective in HLA-B27 positive than negative patients (23.1 vs. 15.2%; *p* = 0.09). HLA-B27-positive patients with enthesitis-related arthritis received biological therapy more frequently than HLA-B27-negative ones (20.4% vs. 0, respectively; *p* = 0.09) (18). Our result corroborate this statement as positive HLA-B27 has an OR = 9.43 (2.27–39.16), *p* = 0.0020. Studies also have reported an increased risk of several systemic autoimmune diseases among relatives of patients with a systemic autoimmune disease (5, 19). Therefore, we looked for family history of autoimmune diseases in the midst of our patients and found a significant match [OR = 9.43 (2.27–39.15), *p* = 0.0020]. Consequently, it is important to ask parents about medical conditions in relatives at the first examination because if there are some, probabilities of inflammatory uveitis, with an underlying autoimmune disease, are higher.

As far as clinical criterion is concerned, uveitis diagnosis under the age of 6 years increased the risk of anti-TNFα therapy in our study [OR = 4.05 (1.16–14.13), *p* = 0.0284]. It is explained by the fact that diagnosis is often delayed (fewer symptoms, less complaints), so inflammation will evolve uncontrolled over a longer period. That is why the severity at onset is increased, complications are already set up, and steroid dependence is higher, which lead to anti-TNFα therapy decision. Logically, ocular complications at the first slit lamp exam are a significant risk factor too [OR = 67.11 (3.78–1191.69), *p* = 0.0042]. Among those complications, macular edema [OR = 11.25 (2.85–43.81), *p* = 0.0005], cataract [OR = 11.87 (2.37–59.49), *p* = 0.0026], and band keratopathy [OR = 12.89 (2.05–80.90), *p* = 0.0064] are the most significant being associated with anti-TNFα therapy; those occur in severest uveitis with chronic inflammation. Furthermore, low visual acuity at diagnosis (≥ 0.3 logMAR) [OR = 11.76 (2.91–47.62), *p* = 0.0005] and especially low binocular acuity at diagnosis (≥0.3 logMAR) [OR = 8.75 (1.93–39.57), *p* = 0.0048] reflect aggressive manifestations of uveitis less steroid response. We also find that having eye surgery [OR = 26.22 (2.63–261.77), *p* = 0.0054] is a significant risk factor; it may consolidate the need of early diagnosis in pediatric uveitis. However, we only had one patient with eye surgery in the control group. This result should be taken carefully. All these factors should be considered as elements of gravity, which must lead to the discussion of an aggressive immunotherapy at the onset of the disease, especially when children show initial eye complications.

Bringing out decision factors for initiating anti-TNFα therapy should help to establish guidelines in pediatric uveitis management. Indeed, children in our study have a mean duration of uveitis evolution before introducing anti-TNFα of 16 months and a mean duration of oral steroid impregnation, during the 5-year follow-up, of 22 months, which is obviously too much. Several studies have shown that anti-TNFα is not only efficient in controlling severe children eye inflammation (8, 12, 13), but it is also safe to use, with a low rate of severe side effects now well-known and treatable (20–24). In the future, we may imagine analyzing these risk factors at the first consultation to estimate the risk of having a severe uveitis, which may need prompt biotherapy. Thus, high-risked children could be monitored more carefully, and anti-TNFα could be introduced sooner, which could allow limitation or reduction of steroid therapy. By doing so, sight damage due to uncontrolled inflammation and to corticosteroid exposition should be reduced, and visual prognoses should be improved. Finally, it may be interesting to design a prospective study to investigate if introducing anti-TNFα sooner can effectively control sight damage.

## Data Availability Statement

The raw data supporting the conclusions of this article will be made available by the authors, without undue reservation.

## Ethics Statement

The studies involving human participants were reviewed and approved by Comité d'Ethique, Université de Strasbourg, France. Written informed consent to participate in this study was provided by the participants' legal guardian/next of kin.

## Author Contributions

AS, DO, and A-CR contributed to conception and design of the study. DO and A-CR organized the database and wrote the first draft of the manuscript. AS and DO performed the statistical analysis. AS, TB, JT, and CS wrote sections of the manuscript. All authors contributed to manuscript revision, read, and approved the submitted version.

## Conflict of Interest

The authors declare that the research was conducted in the absence of any commercial or financial relationships that could be construed as a potential conflict of interest.

## Publisher's Note

All claims expressed in this article are solely those of the authors and do not necessarily represent those of their affiliated organizations, or those of the publisher, the editors and the reviewers. Any product that may be evaluated in this article, or claim that may be made by its manufacturer, is not guaranteed or endorsed by the publisher.
